# Identification of novel nucleotide sequence variations in an extended region of the bovine leptin gene (*LEP*) across a variety of cattle breeds from New Zealand and Nigeria

**DOI:** 10.5194/aab-63-241-2020

**Published:** 2020-07-22

**Authors:** Ishaku L. Haruna, Sibusiso A. Hadebe, Oyekunle J. Oladosu, Ghassan Mahmoud, Huitong Zhou, Jon G. H. Hickford

**Affiliations:** Faculty of Agriculture and Life Sciences, Lincoln University, Lincoln 7647, New Zealand

## Abstract

Leptin is mainly secreted by white adipose tissue in animals. Leptin acts by stimulating or inhibiting the release of a neurotransmitter, which eventually results in a decrease in food/feed intake and an increase in energy expenditure. In this investigation, the polymerase chain reaction (PCR) coupled with single-strand conformation polymorphism (SSCP) analysis was used to reveal nucleotide sequence variations in bovine leptin gene (*LEP*) in 338 cattle of a variety of breeds farmed in New Zealand (NZ) and Nigeria. These included NZ Hereford, Angus, Shorthorn, and crossbred Holstein-Friesian × Jersey cattle and the Nigerian Sokoto Gudali, Red Bororo, White Fulani, and crossbred Holstein-Friesian × White Fulani cattle. Sequence analysis of three regions of bovine *LEP* that encompassed selected coding and non-coding regions, revealed a total of 12 nucleotide sequence variations (six in exons and six in introns). Of these, three are reported here for the first time, whereas nine have been previously described. Some of the variations identified were common in both the NZ and Nigerian cattle breeds, while others were peculiar to particular breeds from a specific region. The sharing of common variants across different breeds irrespective of geography may indicate an evolutionary relationship, just as the differences within a breed might be attributable to either selective pressure for specific traits or random genetic drift. The detection of both new and previously documented variations in bovine *LEP* suggests that the gene is highly variable.

## 1 Introduction

One of the most important factors affecting animal productivity is feed intake. Poor feed intake or inadequate nutrition can affect body weight, growth, reproduction, and milk production, and it can also decrease immunity. Accordingly, a desire to increase animal productivity may require the adoption of measures aimed at increasing or maximizing feed intake. To achieve this, having an understanding of the role of genetics in regulating this important trait is important.

Leptin is a product of the gene that is now called *LEP* but was previously known as *OB*, *OBS*, and *LEPD*. Bovine *LEP* has been mapped to chromosome 4 (Pomp et al., 1997), and it consists of three exons separated by two introns. Exon 1 and part of exon 2 (four nucleotides) are not translated, and only the remaining part of exons 2 and 3 are translated into the functional 16 kDa leptin protein of 146 amino acids in length.

Leptin is considered to be a protein hormone and is mainly secreted from white adipose tissue. This protein is found to regulate feed intake, body weight, immune function, and reproduction (Santos-Alvarez et al., 1999; Kadokawa et al., 2000; Block et al., 2001). Leptin acts on receptors in the lateral hypothalamus to inhibit hunger (Elias et al., 1999) and in the medial hypothalamus to stimulate satiety (Elmquist et al., 1999). It counteracts the effects of neuropeptide Y, a potent hunger promoter secreted by cells in the gut and in the hypothalamus. The hypothalamic release of neuropeptide Y can result in an increase in feed intake and an increase in energy expenditure, among other things (Houseknecht et al., 1998).

Several nucleotide sequence variations have been reported in bovine *LEP*, with these including microsatellite repeat number variation and nucleotide sequence variation (Stone et al., 1996; Konfortov et al., 1999). Konfortov et al. (1999) investigated a 1788 bp portion of *LEP* (comprised of exons 2 and 3 and parts of introns 1 and 2) in 13 different breeds of *Bos taurus* and *Bos indicus* cattle. They reported 20 nucleotide variations, six of which occurred in the exons and at a density of approximately 1 per 84 bp. The density of the intron sequence variations was 1 per 92 bp, giving an overall density of variation of one per 89 bp. This suggests the gene is polymorphic and that if more cattle breeds are examined, more variation might be detected.

The aim of this study was therefore to investigate an extended region of bovine *LEP* in a variety of cattle breeds from New Zealand (NZ) and Nigeria, with the objective of identifying unique and novel variations specific to a given breed or shared across breeds. This would enable improved understanding of the gene and, subsequently, may allow identification of potential gene markers associated with feed intake in cattle.

## 2 Materials and methods

### 2.1 Cattle investigated

Our investigation was carried out in accordance with the Animal Welfare Act 1999 (NZ Government), whether blood was collected in NZ or Nigeria.

A total of 338 cattle from NZ and Nigeria were investigated. The NZ cattle included NZ Hereford (n=23), Angus (n=23), Shorthorn (n=18), and crossbred Holstein-Friesian × Jersey (HF × J-cross) (n=166) breeds; while the Nigerian cattle included Sokoto Gudali (SG; n=18), Red Bororo (RB; n=34), White Fulani (WF; n=32), and crossbred Holstein-Friesian × White Fulani (HF × WF-cross; n=24) breeds. Of these eight breeds, the HF × J-cross and the HF × WF-cross breeds are bred for milk production, whereas the Hereford, Angus, and Shorthorn breeds are farmed primarily for meat production. The Nigerian Sokoto Gudali, Red Bororo, and White Fulani are dual-purpose breeds. Unlike the NZ breeds, which are *Bos taurus* cattle, the Nigerian White Fulani, Red Bororo, and Sokoto Gudali are Zebu (*Bos indicus*) cattle and are characterized by having a fatty thoracic hump on their shoulders, a large dewlap, and being adapted to dry environmental conditions (Mattioli et al., 2000).

### 2.2 Collection of blood samples

Blood samples from each animal were collected (either by piercing the ear or from the tail) onto FTA cards (Whatman, Middlesex, UK) and air dried. DNA from the dried blood was purified using a two-step procedure as described by Zhou et al. (2006).

### 2.3 PCR primer design

Three pairs of primers were designed using DNAMAN Version 5.2.10 (Lynnon BioSoft, Vaudreuil, Canada). These were designed to amplify three regions of the bovine leptin gene (GenBank accession number U50365.1). The primer pair 1 (5${}^{\prime }$-gtctttgaggagatgatagcc-3${}^{\prime }$ and 5${}^{\prime }$-gctgtctttatgccagggg-3${}^{\prime }$) would amplify a 443 bp fragment, consisting of exon 2 and parts of introns 1 and 2; the primer pair 2 (5${}^{\prime }$-agctagtcaggttccacaag-3${}^{\prime }$ and 5${}^{\prime }$-ggttctgcaagggtattcag-3${}^{\prime }$) would amplify a 400 bp fragment of intron 2; and the primer pair 3 (5${}^{\prime }$-ttgctctccccttcctcctg-3${}^{\prime }$ and 5${}^{\prime }$-ctcaggtttcttccctggac-3${}^{\prime }$) would amplify a 430 bp fragment, which consisted of exon 3 and part of intron 2.

### 2.4 Polymerase chain reaction (PCR)

The PCR reactions for all three regions were performed in 15 µL reactions containing the genomic DNA on a 1.2 mm diameter disc of FTA paper, 0.25 µM for each primer, 150 µM for each dNTP (Eppendorf, Hamburg, Germany), 3.0 mM ${\mathrm{Mg}}^{2+}$, 0.5 U of *Taq* DNA polymerase (Qiagen, Hilden, Germany), and 1× the reaction buffer supplied with the enzyme. For each of the three regions, PCR amplification was undertaken in Bio-Rad S1000 thermal cyclers (Bio-Rad, Hercules, CA, USA). The thermal cycling conditions included an initial denaturation at 94 ${}^{\circ }$C for 2 min, followed by 35 cycles of 94 ${}^{\circ }$C for 30 s, 60 ${}^{\circ }$C for 30 s, and 72 ${}^{\circ }$C for 30 s, with a final extension step at 72 ${}^{\circ }$C for 5 min.

### 2.5 Single-strand conformation polymorphism (SSCP) analysis

The SSCP technique was used to detect genetic variation in the amplicons obtained from the PCR reactions. A 0.7 µL aliquot of the amplicons was added to 7 µL of loading dye containing 10 mM EDTA, 0.025 % bromophenol blue, 0.025 % xylene cyanol, and 98 % formamide. The samples were then placed on a hotplate already set at 95 ${}^{\circ }$C for 5 min for denaturation, followed by snap-chilling on wet ice. Samples were then loaded onto 16 cm × 18 cm, acrylamide : bisacrylamide (37.5 : 1) (Bio-Rad) gels. Electrophoresis was carried out using Protean II xi cells (Bio-Rad) for 18 h at 390 V, with a 12 % polyacrylamide gel concentration, and run temperature of 13 ${}^{\circ }$C for amplicon I (part of intron 1, exon 2, and part of intron 2); for 16 h at 390 V, with 10 % polyacrylamide at 18 ${}^{\circ }$C for amplicon II (part of intron 2); and for 24 h at 390 V, with 10 % polyacrylamide plus 4 % glycerol at 15 ${}^{\circ }$C for amplicon III (part of intron 2 and exon 3). The silver staining technique described by Byun et al. (2009) was used to reveal the SSCP banding patterns.

### 2.6 Nucleotide sequencing

Based on the PCR-SSCP patterns identified, cattle that were homozygous with unique banding patterns for amplicons I, II, and III were subjected to direct sequencing at the Lincoln University DNA sequencing facility (Lincoln University, NZ). For these cattle, the amplicons were purified using a MiniElute™ PCR purification kit (Qiagen) and then directly sequenced using the original PCR primers to prime the sequencing reactions in both directions, using the forward and reverse primers.

For rare patterns that were only found in heterozygous form, the unique band was excised from the wet gel and incubated at 69 ${}^{\circ }$C for 1 h as per Gong et al. (2011). A 1 µL aliquot of the incubated product was then mixed with 14 µL of PCR pre-mixture and then amplified using identical conditions to the original PCR reactions.

For each of the regions sequenced, the nucleotide sequences were aligned, and other analyses were undertaken using DNAMAN Version 5.2.10 (Lynnon BioSoft).

## 3 Results

**Figure 1:**
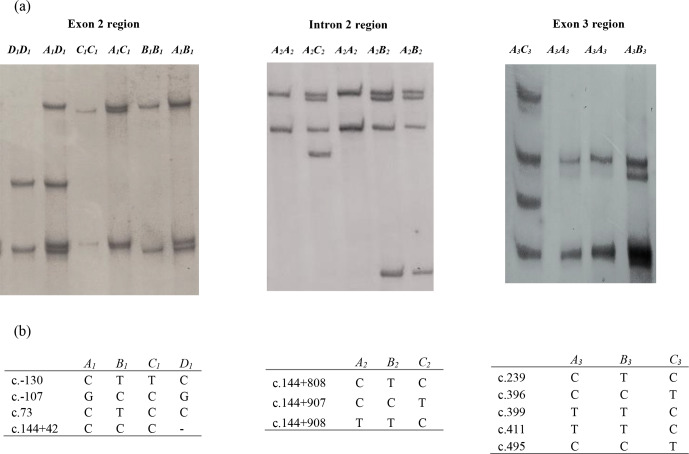
**(a)** Polymerase chain reaction–single strand conformation polymorphism (PCR-SSCP) banding patterns obtained for the three amplicons from the bovine leptin gene. **(b)** Nucleotide sequencing revealed the different sequence variations identified in each of these regions. A dash (–) represents a nucleotide deletion.

In this investigation, the PCR-SSCP technique revealed four banding patterns ($A_{1}$, $B_{1}$, $C_{1}$, and $D_{1}$) in amplicon I (exon 2 and parts of introns 1 and 2), three patterns ($A_{2}$, $B_{2}$, and $C_{2}$) in amplicon II (intron 2 region) and three patterns ($A_{3}$, $B_{3}$, and $C_{3}$) in amplicon III (which spans exon 3 and part of intron 2) (Fig. 1).

Of the four variants identified for amplicon I, the NZ HF × J crossbred cattle had three variants ($A_{1}$, $B_{1}$, and $C_{1}$), and the Nigerian Red Bororo and crossbred HF × WF cattle had three variants each ($A_{1},B_{1}$ and $D_{1})$, while two variants ($A_{1}$ and $B_{1}$) were observed in the NZ Hereford, Angus, Shorthorn, Nigerian Sokoto Gudali, and White Fulani cattle.

In the amplicon II region, all the eight breeds were observed to carry the $A_{2}$ and $B_{2}$ variants, but only the Nigerian Red Bororo had the $C_{2}$ variant.

All eight breeds carried the $A_{3}$, $B_{3}$, and $C_{3}$ variants in the amplicon III region investigated. The frequency of each variant in each region is presented in Table 1.

**Table 1:** Variants and their respective frequencies identified in each of the three amplicons of bovine leptin gene across the NZ dairy and beef and the Nigerian dual-purpose cattle breeds investigated.

Breed	n	Exon 2 region				Intron 2 region			Exon 3 region		
		variants and				variants and			variants and		
		frequencies (%)				frequencies (%)			frequencies (%)		
		$A_{1}$	$B_{1}$	$C_{1}$	$D_{1}$	$A_{2}$	$B_{2}$	$C_{2}$	$A_{3}$	$B_{3}$	$C_{3}$
NZ HF × J (dairy)	166	60.5	39.2	0.3	0.0	92.5	7.5	0.0	66.6	23.8	9.6
NZ beef breeds	64	65.0	35.0	0.00	0.0	67.2	32.8	0.0	62.5	16.4	21.1
Nigerian dual-purpose breeds	108	54.2	29.2	0.0	16.6	62.0	19.0	19.0	60.6	20.8	18.6

A total of 12 nucleotide variations were identified across the three regions examined. Eleven were nucleotide substitutions: c.-107G/C and c.-130C/T located in intron 1; c.73C/T (p.Arg25Cys) in exon 2; c.144+808C/T, c.144+907C/T, and c.144+908T/C identified in intron 2; and c.239C/T (p.Ala80Val), c.396C/T (p.Gly132=), c.399T/C (p.Val133=), c.411T/C (p.Ala137=), and c.495C/T (p.Pro165=) located in exon 3. The remaining nucleotide variation was a deletion (c.144+42delC), located in intron 2.

**Figure 2:**
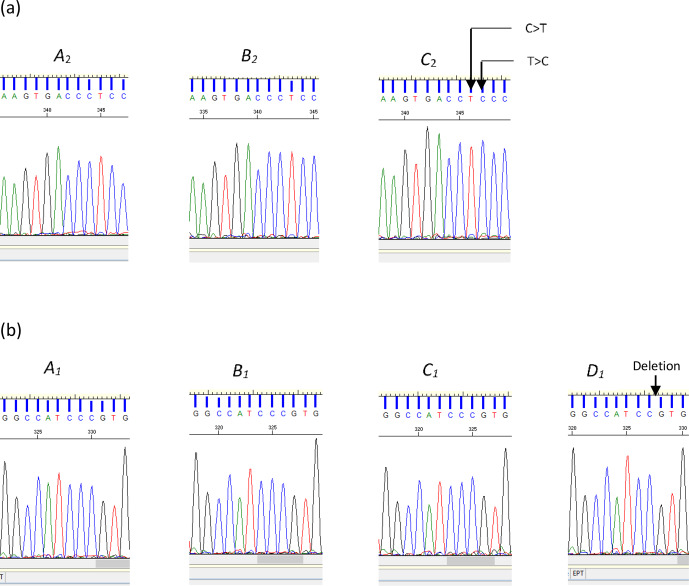
**(a)** Sequence chromatograms illustrating the differences between variants $A_{2}$ and $B_{2}$ and variant $C_{2}$ of the bovine *LEP* intron 2 region. The chromatogram of variant $C_{2}$ indicates the positions of the two nucleotide substitutions C>T and T>C (nucleotide sequence variation c.144+907C/T and c.144+908T/C respectively). **(b)** Chromatograms for $A_{1}$, $B_{1}$, $C_{1}$, and $D_{1}$. The chromatogram of variant $D_{1}$ illustrates the position of the deleted cytosine (c.144+42delC).

Of them 12 variations identified across the three regions examined, the deletion (c.144+42delC) and two substitutions (c.144+907C/T and c.144+908T/C) were novel and are reported here for the first time (Fig. 2). The remaining nine variations, with their respective reference SNP (rs) numbers have been previously reported to Ensembl (EMBL-EBI, Hinxton, United Kingdom, release 96 – April 2019, ARS-UCD1.2 for the cow leptin gene).

**Figure 3:**
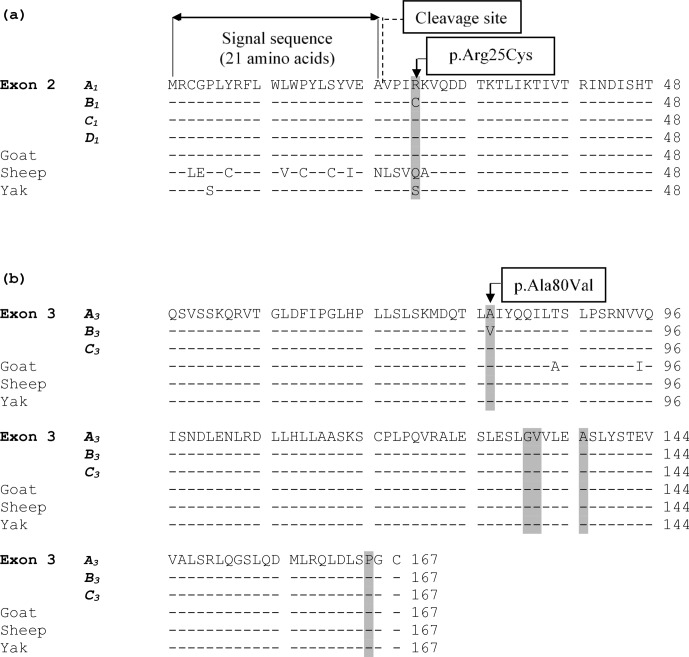
**(a)** An alignment of the predicted amino acid sequence of leptin protein obtained from the four variants ($A_{1}$, $B_{1}$, $C_{1}$, and $D_{1}$) identified in exon 2. The grey highlighted areas mark the position of the amino acid difference between the variant sequences and other species. **(b)** Alignment of the putative amino acid sequence of leptin protein from the three variants ($A_{3}$, $B_{3}$, and $C_{3}$) in exon 3 and from sequences in other species. The grey highlighted areas mark the position of amino acids where the underlying nucleotide sequence is variable. The symbol “–” represents the same amino acid as the top (reference sequence). The amino acid sequence for cow was obtained from the coding sequences examined in this study, whereas the others were obtained from GenBank JQ739233.1 (goat), MH716186.1 (sheep), and EU603265.1 (yak).

The alignment of the predicted amino acid sequence of leptin protein obtained from the four variants ($A_{1}$, $B_{1}$, $C_{1}$, and $D_{1}$) in exon 2 and three variants ($A_{3}$, $B_{3}$, and $C_{3}$) in exon 3 are given in Fig. 3.

## 4 Discussion

Recently, there has been some interest in leptin gene variations in livestock, mainly as a consequence of their potential for use as gene markers for improving the efficiency of selection for quantitative traits.

Of the 12 variations identified in this study, three are novel, and nine have been described previously. Of the nine reported previously, two would if expressed, change the amino acid sequence of the protein. These are c.73C/T (p.Arg25Cys) in exon 2 and c.239C/T (p.Ala80Val) in exon 3. The p.Arg25Cys substitution would result from the C/T substitution located 73 bp from the start of exon 2 in a region that is proximal to the signal sequence of leptin and that has been described previously (Konfortov et al., 1999; Buchanan et al., 2002; Lagonigro et al., 2003). The signal sequence contains 21 amino acids and it is cleaved off before leptin is excreted from adipose tissue (Zhang et al., 1997). The predicted cleavage site of the signal sequence is C terminal to an alanine at position 21 (Zhang et al., 1997). The p.Ala80Val would result from a C/T substitution located 95 bp from the start of exon 3. It has also been described previously (Konfortov et al., 1999; Haegeman et al., 2000).

Zhang et al. (1997) suggested that the p.Arg25Cys change is situated in the first of the four α helices of the leptin protein and at a location that varies considerably between species. They reported glutamine in primates and mice, arginine in dogs, tryptophan in pigs, and histidine in rats. Therefore, it is likely that the p.Arg25Cys change is allowed because it does not have functional significance. This view was also shared by Konfortov et al. (1999). It is also conceivable that this position may be polymorphic in other species. This is supported by the amino acid alignment of leptin protein from different ruminant species (Fig. 3), with position 25 of the protein in goats being an arginine residue, while sheep have glutamine, and yak have serine. Variation at this position therefore appears to be tolerated. Since arginine and cysteine have alkaline and neutral side chains respectively, this variation could be considered a nonconservative substitution.

However, Buchanan et al. (2002) hypothesized that the p.Arg25Cys change has a functional effect on the leptin molecule. The paper suggested that the presence of cysteine in the α helix of the leptin molecule may disrupt the binding of leptin to its receptor. This is supported by the observation that the leptin receptor contains a conserved trough typical of haemopoietic cytokine receptors, into which the A and D helixes of haemopoietic cytokines dock (Sprang and Bazan, 1993). They argued that a change between two very different amino acids, such as arginine and cysteine at this location, may have deleterious effect on this process. Also, their report suggests that the presence of another unpaired cysteine in the leptin molecule could destabilize the disulfide bridge found between the two existing cysteines, thus, affecting the structure of the leptin molecule and, consequently, its function (Rock et al., 1996). Studies have shown that this disulfide bridge is critical for biological function (Rock et al., 1996; Zhang et al., 1997).

In comparison, the p.Ala80Val polymorphism is located in a conserved region of the leptin protein (Komisarek and Dorynek, 2005). The c.396C/T (p.Gly132=), c.399T/C (p.Val133=), c.411T/C (p.Ala137=), and c.495C/T (p.Pro165=) nucleotide sequence differences in exon 3 are all synonymous and occur in a conserved region of leptin.

Three nucleotide changes, which included a deletion (c.144+42delC) and two substitutions (c.144+907C/T and c.144+908T/C) occurred in the intron 2 region. They are reported here for the first time. In amplicon I, the $D_{1}$ variant, carrying the deletion c.144+42delC was identified only in the Nigerian Red Bororo and the crossbred Holstein-Friesian × White Fulani cattle breeds. In amplicon II, the $B_{2}$ variant, with the haplotype c.144+808C, c.144+907T, and c.144+908C was found in all the NZ and Nigerian breeds investigated, whereas the $C_{2}$ variant for amplicon II, which carries the substitutions c.144+808T, c.144+907C, and c.144+908T was only detected in the Red Bororo breed. The occurrence of variant $B_{2}$ with GenBank accession number MN082389 in all the nine breeds (NZ and Nigerian cattle breeds) investigated suggests that this variation is shared across cattle of *Bos taurus* and *Bos indicus* (African Zebu cattle) origin. This result is not surprising, given the close relationship between *Bos taurus* and *Bos indicus* in terms of origin. There are suggestions that these two species originated from a common ancestor, and the wild auroch, *Bos primigenius*, is the progenitor of all taurine and zebu (African *Bos indicus*) cattle (Edwards et al., 2007). Estimates of divergence times for *Bos taurus* and *Bos indicus* from a common ancestor are all pre-Neolithic and range from approximately 2 million to 330 000 years ago, depending on the genetic markers and the calibration of the evolutionary molecular clock (Hiendleder et al., 2008). There are also some reports that suggest the *Bos indicus* populations may have been produced at a later date through breeding and selection from *Bos taurus* cattle (Epstein, 1971; Epstein and Mason, 1984). Either way, each of these aforementioned studies highlighted the evolutionary link between *Bos taurus* and *Bos indicus* cattle. The sharing of common haplotypes suggest that cattle studied here might be more closely related, although it does not provide definitive proof. The suggestion is supported by Dunner et al. (2003) who suggested that the pattern of haplotype sharing is an indicator of the history of the different bovine breeds, and thus the distribution of shared haplotypes is useful in describing population relationships.

In the amplicon I region investigated, the $D_{1}$ variant carrying the novel deletion c.144+42delC was proximal to the putative splice-donor site. The deletion variant was only identified in the Nigerian Red Bororo cattle and the crossbred Holstein-Friesian × White Fulani-cross cattle. This suggests that this variation may only be found in the African Zebu cattle, since it was not observed in the NZ HF × J-cross cattle or the other NZ beef breeds. The occurrence of this variation around the exon/intron boundary may possibly have functional implications for gene expression and protein assembly. To illustrate this point, Sjakste et al. (2011) investigated variation in *MSTN *in Latvian Darkhead sheep and reported nucleotide sequence variations (c.373+18G/T and c.373+101C/T) at the splice-donor site and showed that the G nucleotide in the c.373+18 position, initiated single-strandedness in the first CUG repeat and G triplet location. They further suggested that hairpin-loop development in that region could be followed by rearrangement of the spatial topology between the two other CUG repeats. Such perturbation of the pre-mRNA secondary structure could potentially influence sequence interaction with different regulatory proteins and the efficiency of lariat formation, and this could then potentially affect transcription and splicing efficiency (Chasin, 2007; Hiller et al., 2007; Aznarez et al., 2008). There is also evidence suggesting that introns are functionally active participants in gene and genome functionality, as they can encode regulatory elements (Yutzey et al., 1989) that participate in splicing, transcription, and recombination events. It will therefore be interesting to further investigate the effect of this deletion in the Red Bororo and Holstein-Friesian × White Fulani-cross cattle.

In the amplicon I region examined, the $C_{1}$ variant (carrying the nucleotide sequence variations c.-107C and c.-130T) had the lowest frequency, which was also reflected at the genotype level. Again, this is not surprising, especially considering the fact that this variant was only identified in one heterozygous sample ($A_{1}C_{1}$) out of the 166 samples of NZ HF × J-cross cows investigated. Since this variant had very low frequency in the cattle studied here, it may in future be difficult to ascertain how it affects the function of the gene. The investigation of more cows and more breeds may also resolve this issue, especially as in the Ensembl report on Iranian *Bos taurus* from the NextGen Project (NextGen Consortium, 2020) suggests the G and C nucleotides in c.-107G/C (rs29004485) had a frequency of 69 % and 31 % respectively (remapped to ARS-UCD1.2; release 150), and the C and T alleles in the nucleotide variation c.-130C/T (rs29004484) had a frequency of 69 % and 31 % respectively.

## 5 Conclusion

The detection of new as well as previously documented variations in bovine leptin gene indicates a possibility of identifying potential gene marker(s) for the selection of specific traits for increased efficiency in animal production. Some variants were common across the *Bos taurus* and *Bos* *indicus* species, which supports the theory of a possible descent from a common ancestor.

## Data Availability

Data are available upon request to the corresponding author.
